# Cases Illustrating the Treatment of Badly-United Fractures of the Thigh

**Published:** 1855-11

**Authors:** Gurdon Buck

**Affiliations:** Surgeon to the New York Hospital


CASES ILLUSTRATING THE TREATMENT OF BADLY-UNITED FRACTURES OF THE THIGH. By GURDON BUCK, M. D., Surgeon to the New York Hospital. Read April 4, 1855.

*Case I.*—Jules Cæsar, seaman, native of France, age 19 years, fell from aloft on the 27th of March, 1849, and fractured his left thigh near the middle. On the 3d of May he was received into the New York Hospital, into Ward No. 7 of the 2d Surgical Division. Union had taken place, yet a slight degree of motion was still perceptible at the fracture. The overlapping ends of the fragments bowed outwards, and were surrounded with considerable callous deposit. By careful measurement the limb was found to be two inches and a quarter shorter than its fellow. On the 9th of May, six weeks after the injury, the thigh was refractured in the following manner:

Jarvis' Adjuster was first applied, and the thigh put powerfully upon the stretch, as is done in reducing a luxation of the hip. The thigh being then seized at the knee and below the hip, was forcibly bent over the knee of the operator in every direction, so as to liberate the fractured ends, and disengage them from each other.

The rupturing of the adhesions was audible to all present. The patient had obstinately refused to be etherized; yet he did not complain of severe pain during or after the operation. The limb could now be brought down to its full length with the hands alone; when left to itself, it resumed its original shortened condition. Without delay the limb was bandaged and put up in the straight apparatus, and extension kept up as constantly as the patient could bear. At the end of three weeks firm union had taken place and the difference in length was reduced to less than one inch.

After another three weeks the limb was transferred to the double inclined plane, and passive motion, together with frictions, employed to overcome the rigidity of the knee-joint, produced by the long confinement and inaction of the limb.

On the 17th of August, fourteen weeks after the refracture, when the patient took his discharge, he could walk well with the aid of a cane, the shortening of the limb being still less than one inch. In treating this case we had not yet the advantage of the improved mode of keeping up constant extension, which has since been brought into use in the hospital.

*Case II.*—John McKee, laborer, native of Ireland, age 19 years, admitted into the New York Hospital on the 11th of October, 1851, with fracture of the os femoris at its middle, caused by being struck with a fragment of rock from a blast.

An external wound over the seat of fracture, supposed to communicate with it, deterred from the employment of any active measures for keeping up extension.

On the 4th of December, nearly eight weeks after the injury, it was found that partial union only had taken place. The limb was shortened two inches, and considerably bowed outwards at the seat of fracture. The external wound having closed, it was determined to refracture the limb, and subject it to constant extension by a new and improved method, which consisted in applying to either side of the limb, and in contact with the surface, a band of adhesive plaster of double thickness, three inches in breadth, and long enough to extend from the seat of fracture to ten inches below the foot. The lower ends of the bands being overlapped and stuck together, formed a loop, within which a square block of wood was placed. This block served as a point from which traction was to be made. Over the bands a roller was to be applied in the usual way, from the toes upwards. This contrivance permitted constant extension to be kept up, without pain or excoriation, or sloughing of the skin.

Its advantages in the treatment of primary fractures of the thigh had been fully tested in practice at the hospital, and its application to cases like the present, where so much depended on maintaining constant extension of the limb to the utmost degree, it was thought would be peculiarly advantageous.

Instead of Jarvis' Adjuster, the ordinary long splint was used in this case to put the limb upon the stretch.

As in the preceding case the thigh, while on the stretch, was bent in every direction, till all the adhesions were ruptured, and the difference in length reduced to three-fourths of an inch. From the sensation perceived during the operation, it was evident that the uniting material was chiefly callus.

The new method of extension was forthwith resorted to in connection with the straight apparatus.

At the end of eight weeks the apparatus was removed, and the fracture found firmly united, the shortening of the limb being less than an inch.

When discharged from the hospital, on the 27th April, patient walked about on crutches, and was gradually regaining the use of his limb.

*Case III.*—On the 25th of January, 1853, Richard Williams, a seaman, 21 years of age, was admitted to the hospital, with fracture of the right thigh, near the junction of its upper and middle third, that had happened by falling from aloft 16 days prior to his admission. But little swelling remained about the seat of fracture. The shortening of the limb amounted to two inches and three-quarters.

The limb was put up in the straight apparatus, and constant extension by the new method employed, as in Case No II., with the view of stretching the callus without rupturing it.

After the expiration of three weeks it was found that a difference of two inches in the length of the limb still existed, notwithstanding extension had been kept up to the full degree. Five weeks having now passed since the injury, and so little having been gained by stretching the callus, it was determined to rupture it, as in the preceding cases. This was done on the 14th of February, with the aid of ether, and had the effect of reducing the difference in length to one inch. The previous treatment was at once resumed, and continued for thirty-one days. On removing the apparatus firm union had taken place, with a shortening of scarcely one inch.

*Case IV.*—John Healy, a seaman, native of England, aged 19 years, admitted on the 17th of February, 1854, into Lodge A. in the New York Hospital, with injuries that had happened eight weeks ago in consequence of a fall from a loft, from a height of about sixty feet. The right thigh had been fractured near the middle, and united with great deformity of the limb. Short splints having been applied to the thigh by the mate of the vessel, and the limb below the knee left unsupported, the leg, with the lower fragment of the femur, had rotated outwards, and union had taken place in this position, so that the foot rested on its outer edge as the patient lay on his back with his limb extended. An exuberant deposit of callus surrounded the fractured ends, and no motion was perceptible at the seat of fracture. The absolute shortening of the limb could not be ascertained, for a reason that will appear in the sequel. Besides the fracture of the thigh, the left hip had also sustained a serious injury. The limb lay powerless, and was rotated outwards. The trochanter major was drawn higher up towards the crest of the ilium than natural, as was evident from a comparison of the two sides. The limb could be rotated with equal facility inwards and outwards, and crepitus could be felt with the hand grasping the trochanter. Severe pain in the joint was produced by these manipulations. By extending the limb it could be lengthened one inch, but, when left to itself, it resumed its original length, which was found, on measurement, to be one-fourth of an inch shorter than the right limb. The existence of a fracture through the neck of the femur, still ununited, being thus ascertained, it became a matter of the utmost importance to our unfortunate patient to rectify, if possible, the distorted condition of the right limb; for it was found that he had no power when assisted to stand up, to bear any weight on his left leg, and he could only support himself on his right leg by throwing himself far over to the right side. It was therefore determined to refracture the right thigh, and treat it *de novo.*

This was done on the 20th of February, with the aid of Jarvis' Adjuster, the patient being previously etherized.

Immediately after the operation the limb was put up in the straight apparatus, and a moderate degree of extension only kept up. Special care, however, was taken to maintain the limb in its true bearings. The left limb was at the same time placed on the double inclined plane. After six weeks the apparatus was removed from the right limb, and solid union found to have taken place, the position and bearings of the limb being natural.

One week later the left limb was removed from the inclined plane, and, on examination, crepitus could no longer be felt at the hip, nor any elongation produced by extension, nor pain excited by rotation of the limb. The trochanter still appeared more salient and higher up on the pelvis than natural. The right leg was half an inch longer than the left.

At the time of his discharge from the hospital, the 5th of July, the patient, who had steadily improved, was able to walk without crutches, supporting himself erect on both limbs alike.

*Case V.*—John Land, a Norwegian seaman, aged 29 years, of a robust constitution, was admitted on the 19th of May, 1854, into Lodge C., New York Hospital, with fracture of the lower jaw and left arm, besides a fracture of the left thigh at its middle. He had fallen from aloft at sea, a height of about forty feet. Though five weeks had passed since the accident, no bony union had taken place in either of the fractures. They were still very movable, though an exuberant deposit of callous material surrounded the ends of the fragments, both of the arm and thigh. The shortening of the thigh amounted to four inches, with extensive overlapping of the fragments at the seat of fracture. This deficiency of bony union was, no doubt, to be attributed to a cachectic condition of the patient, induced by the privations and sufferings he had endured since the accident. Restricted by the condition of his jaw to the use of liquid nourishment, and only scantily supplied with such as was suitable, he had suffered much of the time from hunger; his body had become emaciated, and his complexion sallow and anemic.

Our first care was to renovate his impoverished system by an abundant supply of nutritious food, in a liquid form, and also by the administration of tonics. Soon after his admission an extensive abscess developed itself at the middle of the left leg, on its outer surface, and was followed by sloughing of a considerable portion of the superficial muscles. This complication rendered it impossible to employ any means of reducing the amount of shortening. Eleven weeks more passed before the abscess healed and the limb regained a condition that would admit of any attempt to improve it. In the mean time, with restored health, bony union had taken place in the fractures, and the patient's deformity had become permanent for life, unless surgical art could interpose for his relief. Though sixteen weeks had now transpired since the original injury occurred, it was determined to refracture the femur, and endeavor, by constant extension, to reduce the amount of shortening. On the 6th of August the operation of refracturing was performed, the patient being first subjected to the influence of ether. While powerful extension was kept up by means of Jarvis' Adjuster, the thigh was flexed in every direction, and the union between the ends of the fragments broken up. The shortening was now reduced from four inches to one and a quarter.

The straight apparatus, with long extending bands of adhesive plaster, was applied to the limb, and special care taken to keep up the extension to the utmost degree during the subsequent treatment.

At the expiration of 44 days solid union had taken place, with a shortening of one inch and three-eighths.

The rigidity of the knee and ankle joints, and the thickening and swelling of the cellular tissue, consequent on the preceding inflammation and long-continued inaction of the limb, very much retarded the restoration of its functions. He has, however, been gradually improving, and has the prospect of regaining, in a good degree, his former activity.

*Case VI*—Is that of a boy, 5 years old, still under treatment at the New York Hospital, by my colleague, Dr. T. M. Halsted, who has kindly permitted me to make use of the case.

He was admitted into Ward No. 2 of the First Surgical Division on the 3d of February, 1855, with a fracture of the left femur, that happened about four months previously, from a fall from off a front stoop. The limb was put up with a simple anterior and posterior splint by a bone-setter. The result at the end of four weeks not being satisfactory the limb was refractured and again put up in the same manner. At the end of another four weeks it had again united with deformity, and was refractured a second time. In the same manner the limb was again refractured for the third time; after which the patient was sent to the hospital. When admitted the limb presented a great degree of deformity. The upper fragment, which consisted of the superior third of the bone, overlapped the lower anteriorily and to the outside, with a very marked bowing outwards, and a shortening of two inches and three quarters by accurate measurement. The union, though quite firm, permitted a slight degree of motion at the seat of fracture.

The limb was now put up in the straight apparatus, and constant extension applied by means of adhesive bands in the hope of stretching the callus. Counter pads were also adjusted near the seat of fracture to overcome the bowing. This plan was continued for 27 days, till the 2d of March, when it was found that the shortening had only been reduced to 2 inches. It was therefore decided, in consultation, to refracture the bone once more, and for the fourth time. This was done with the aid of ether by manipulation alone, and without stretching the thigh. The limb was again put up as before in the straight apparatus, and, on applying extension, was brought down to within one-quarter of an inch of its natural length.

On the 31st of March, after the treatment had been continued four weeks, the limb was examined and union found solid, with a shortening not exceeding one-fourth of an inch.*

*Remarks.*—Some of the causes of deformed union after fracture, are illustrated by the above cases.

In the absence of surgical aid at sea, for example, the fracture remaining unreduced, the unresisted contraction of the muscles draws the ends of the fragments of bone past each other, producing a shortening of the limb, which, if allowed to continue, becomes permanent. The same result happens where the fracture is complicated by other injuries, or by coexisting diseased conditions of the limb itself, such as erysipelas, abscess, sloughing, etc., making it impossible for the surgeon to secure for the limb a proper condition, or apply the fixtures indispensable to maintain it in a state of reduction. The intractability of the patient, or a state of mania or delirium may thwart the best-devised plan of treatment, and produce the same result. Removing the splints and apparatus from the fractured limb too soon, and premature attempts to use it, are also sure to produce deformity.

The deformity thus resulting may be the shortening of limb to a greater or less degree, without any deviation from its natural bearings. The minimum amount of shortening to be regarded as a deformity requiring or warranting surgical interference, must depend mainly on the inconvenience experienced by the patient himself. It can hardly be rated at less than one inch and a half, though with the present improved mode of keeping up constant extension by adhesive plaster, the attempt may be made under favorable circumstances to diminish even that amount of shortening.

* This patient, when discharged from the hospital on the 29th of May, was able to walk without crutches, the fractured limb being still only one-fourth of an inch shorter than its fellow.

Besides shortening of the limb there may be bowing outwards or inwards at the seat of fracture. There may be also an outward rotation of the limb on its axis below the fracture. The effect of this is to change entirely the bearings of the limb, which is strikingly illustrated in Case IV.

Accident must have early suggested to surgeons the mode of treating this class of deformities. Patients, in their first attempts to resume the use of a fractured thigh or leg, frequently refracture the newly united bone by accidental falls, and this is generally followed by a speedy reconsolidation of the fracture; sometimes this has proved an advantage by enabling the surgeon to correct a defect in the original cure. This was illustrated in a recent case at the hospital. A young man, 17 years of age, was treated during the summer of 1854 for a compound fracture of the left thigh near the middle. The wound complicating the fracture became the seat of hospital gangrene, and prevented the application of any extension of the limb. The result was a shortening of one and a half inches at the date of his discharge on the 3d of October. On the 25th of the same month, and nearly sixteen weeks after the first fracture, he was admitted again with a refracture caused by an accidental fall. There being now no external wound to interfere with the treatment, constant extension was applied, and speedy reconsolidation effected, with only half an inch shortening.

When the interval since the fracture is not too long, and the ends of the fragments are still movable, extension alone may serve to elongate the limb sufficiently. If, however, after a few days' trial, no material advantage has been gained by stretching the callus, there should be no longer delay in rupturing it. The method of operating employed in the above cases has proved safe, and it is believed it may be resorted to with very little risk of injury to the soft parts. By keeping the limb powerfully on the stretch while flexion is made the ends of the fragments cannot be protruded far towards the surface in any direction. The inflammation and swelling following the operation, if any, are so slight as not to interfere with the immediate application of the apparatus for maintaining extension.

An important practical question in relation to this subject is, what interval of time may elapse within which it is admissible to resort to this mode of treatment? Guided solely by the consideration of the time required by the callus to become consolidated, we should be restricted in the application of our treatment to much narrower limits than clinical experience would impose. There is considerable discrepancy among authorities on this point, their views being influenced by the mode of treatment which they advocate. Some advise extension alone, with the view of stretching the callus, and discard altogether the operation of rupturing.

Dupuytren, who advocated extension without rupture, fixed the sixtieth day as the ordinary limit, though he admitted that, in exceptional cases, it might be considerably exceeded. Æsterlin (cited by Laugier, *Thése sur les cals difformes*, etc., etc., Paris, 1841), an advocate of refracture, places the limits at six months to a year for adults, and at a longer period for children. Professor Velpeau (*Médecine Opératoire*, 2d edition, T. 1, pp. 604, 605), divides deformities consequent on fracture into two classes: 1st. Where there is shortening alone of the limb, with overlapping of the fragments. 2d. Where there is angular deformity.

In the first case he thinks that refracture should not be attempted beyond the third month, for fear of fracturing the bone elsewhere; or, in case of separating the fragments, their cicatrized extremities should fail to reunite after being brought to confront each other by extension.

Our Case No. V. is an example of successful refracture beyond the limits prescribed by Velpeau, and the subsequent rapid reconsolidation of the fragments show that the fears of non-union are not always confirmed in practice.

There is still less ground for fear of non-union, if we consider that, after refracture of a callus of so long standing, it is seldom practicable, and never necessary, to employ extension to such a degree as to confront the ends of the fragments. If the amount of shortening be reduced to one inch, or even one inch and a half, there still remains sufficient overlapping of the newly ruptured surfaces to effect a union, and also to secure to the patient a useful limb, with little or no halting in his gait.

In his second class of callus with angular deformity, Professor Velpeau advises refracture of the callus at any period of its existence, however remote, and relates a case of an adult patient with an oblique fracture of the femur, that had united with a shortening of five inches, who, by an accidental fall, refractured the callus ten months afterwards, and recovered in two months, with only one inch shortening.—*Transactions N. Y. Academy of Med.*

